# DISPARITIES IN HEARING LOSS AMONG OLDER ADULTS: HEALTH AND RETIREMENT STUDY, 2016–2018

**DOI:** 10.1093/geroni/igae098.2528

**Published:** 2024-12-31

**Authors:** Nasya Tan, James Pike, Lindsay Kobayashi, Philippa Clarke

**Affiliations:** University of Michigan, Ann Arbor, Michigan, United States; New York University, New York, New York, United States; University of Michigan, Ann Arbor, Michigan, United States; University of Michigan, Ann Arbor, Michigan, United States

## Abstract

As hearing loss becomes more prevalent in middle and older ages, more research is needed to identify factors associated with hearing loss among this population using nationally representative data. This study aimed to examine associations between sociodemographic factors and hearing loss using 2016-18 data from the Health and Retirement Study, a population-based cohort of US adults aged 51+ (n=17,468). Respondents self-reported hearing loss, age at assessment, gender, race/ethnicity, and education. We ran weighted logistic regression models that accounted for the complex survey design, stratified by gender and race/ethnicity. Covariates included year of assessment and birth cohort. Results showed that for every 5-year increase in age, the odds of hearing loss increased by 40% (95%CI=1.26, 1.55). Compared to White males, Black males were 42% less likely to have hearing loss (95%CI=0.44, 0.77). Compared to White females, Black females were 46% less likely to have hearing loss (95%CI=0.42, 0.70). Among men, those with less than a high school education were 23% less likely to have hearing loss (95%CI=0.63, 0.94)than college graduates. However, men with an associate’s degree or some college education were 34% more likely to have hearing loss (95%CI=1.14, 1.58). Education was not associated with hearing loss among women. This study highlights disparities in hearing loss in later life by race/ethnicity, gender, and education. It adds to the current literature by identifying disparities in hearing loss, and is the first study to examine hearing loss disparities using nationally representative data on middle-aged and older adults in the Health and Retirement Study.

